# A safe and easy-to-use ultrasound-guided hydrodissection technique for the carpal tunnel syndrome: a minimally invasive approach

**DOI:** 10.1007/s40477-021-00597-5

**Published:** 2021-07-02

**Authors:** Thomas Mathieu, Els Lemmens, Gaëtane Stassijns

**Affiliations:** 1 Faculty of Medicine and Health Sciences, University of Antwerp, Antwerp, Belgium; 2 Department of Physical and Rehabilitative Medicine, Antwerp University Hospital, Wilrijkstraat 10, 2650 Edegem/Antwerp, Belgium; 3 Sports Medicine Centre ‘de Merode’, Turnhout, Belgium

**Keywords:** Carpal tunnel syndrome, Hydrodissection, Flexor retinaculum, Median nerve, Entrapment neuropathy

## Abstract

Carpal tunnel syndrome (CTS), compression of the median nerve lying deep under the flexor retinaculum, is the most common entrapment neuropathy of the upper limb. After a failure of conservative treatments, such as non-steroidal anti-inflammatory drugs (NSAIDs) and splinting, interventional techniques are required. Hydrodissection is an injection technique that separates the nerve from the surrounding tissue. Although this technique is gaining ground in modern medicine, the state-of-the-art literature is lacking a clear protocol or approach for hydrodissection for CTS. In this article, we describe a safe, minimally invasive, effective, and easy-to-use ultrasound-guided hydrodissection technique for CTS.

## Introduction

Carpal tunnel syndrome (CTS) is caused by compression of the median nerve lying deep under the flexor retinaculum at the wrist level and is the most common entrapment neuropathy in the upper limb [1]. Conservative treatments, such as removing causative activities, splinting, treating the underlying disease, non-steroidal anti-inflammatory drugs (NSAIDs), physical therapy, and occupational therapy, are the first steps of treatment. When conservative measures fail, interventional injection techniques are considered as the next step. Landmark-based or blind injection and ultrasound-guided injection of the carpal tunnel are often used in musculoskeletal practice. Many approaching methods for carpal tunnel injection have been described. Evers et al. stated that ultrasound-guided injections are more effective than blind injections in the treatment of CTS [2].

Nerve hydrodissection is one of the upcoming techniques of musculoskeletal ultrasound procedures [3]. Hydrodissection is an injection technique that separates the nerve from the surrounding tissue. In this study, we have developed a new ultrasound-guided procedure of mobilizing the median nerve away from the deep surface of the flexor retinaculum by percutaneous hydrodissection. We found the new technique to be safe, effective, and easy to use if the physician is experienced with ultrasound-guided infiltrations. Our goal was to develop a minimally invasive technique that could be easily used by a physician experienced in ultrasound-guided injections but with no experience in hydrodissection.

## Anatomy

The carpal tunnel is an osteofibrous canal located in the volar wrist. The boundaries are the carpal bones forming the floor, and the flexor retinaculum forming the roof. The median nerve runs in the distal forearm between the flexor pollicis longus tendon (FPL) and the flexor digitorum superficialis tendon (FDS). Going towards the distal end, the median nerve becomes superficial, reaches the carpal tunnel, and travels through it in a superficial position just below the flexor retinaculum. Here it usually travels superficially to emerge between the tendons of the FPL and FDS to the index and middle fingers [4, 5].

The flexor retinaculum is the superficial roof of the carpal tunnel. The retinaculum is about 3–4 cm wide and inserts into the scaphoid tuberosity and the pisiform (proximal carpal tunnel) and subsequently into the trapezium and the hook of the hamate (distal carpal tunnel). Compression of the median nerve against the inferior face of the flexor retinaculum results from increased pressure inside the carpal tunnel [5].

## Technique

**Fig. 1:**
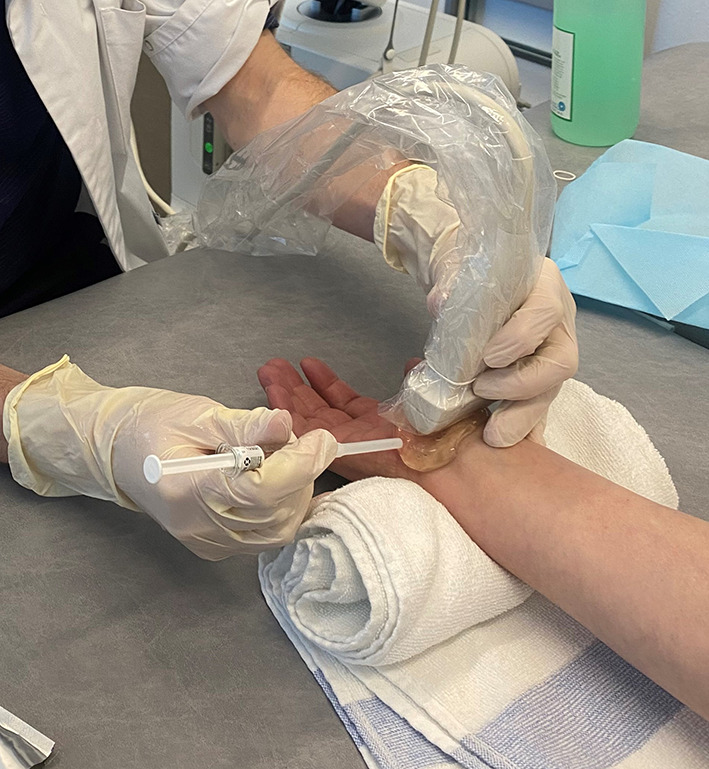
Overview of the positioning of the patient and the needle before the needle is inserted

**Fig. 2:**
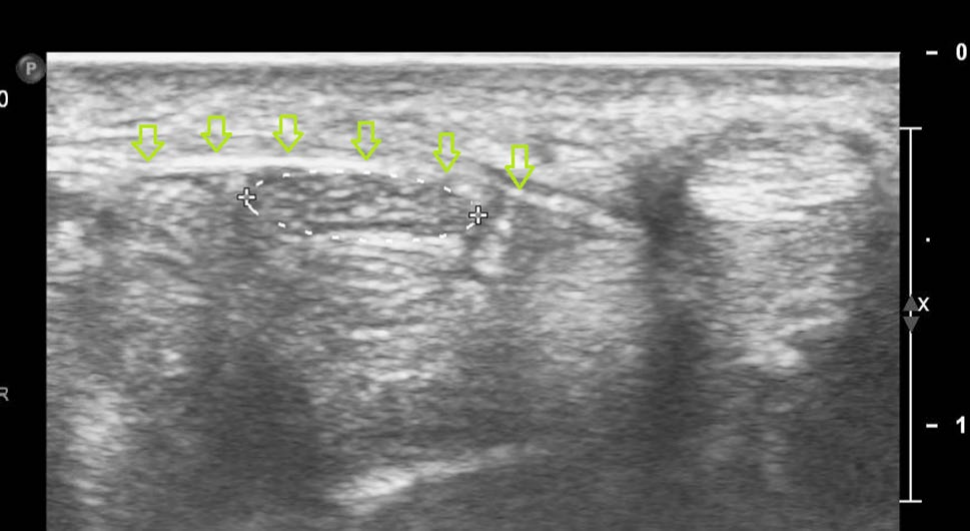
Ultrasound image of the carpal tunnel in the axial plane. *Legend:* void arrows: flexor retinaculum or transverse carpal ligament; circle in dotted line: median nerve

**Fig. 3:**
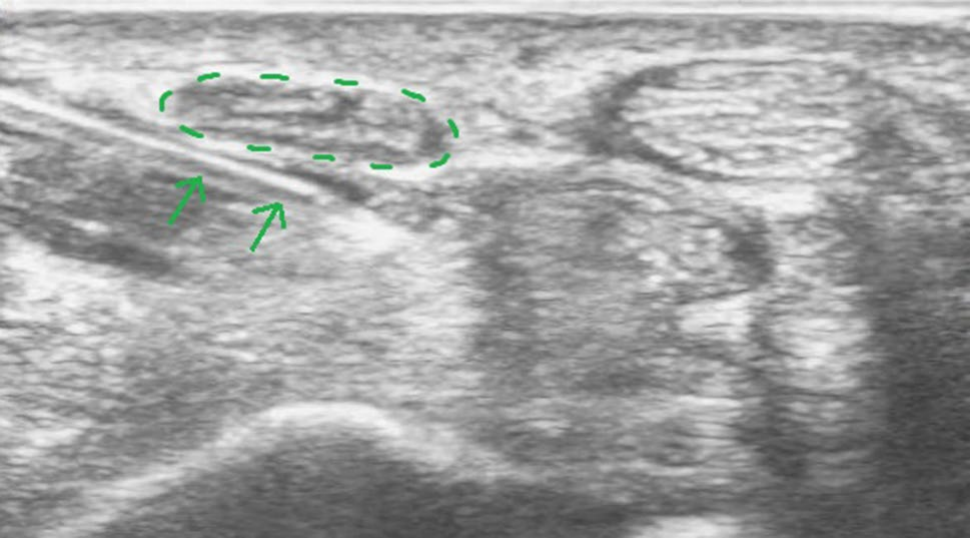
Ultrasound image of the carpal tunnel in the axial plane during Step 4 of the hydrodissection. *Legend:* circle in dotted line: median nerve; arrow: injection needle

**Fig. 4:**
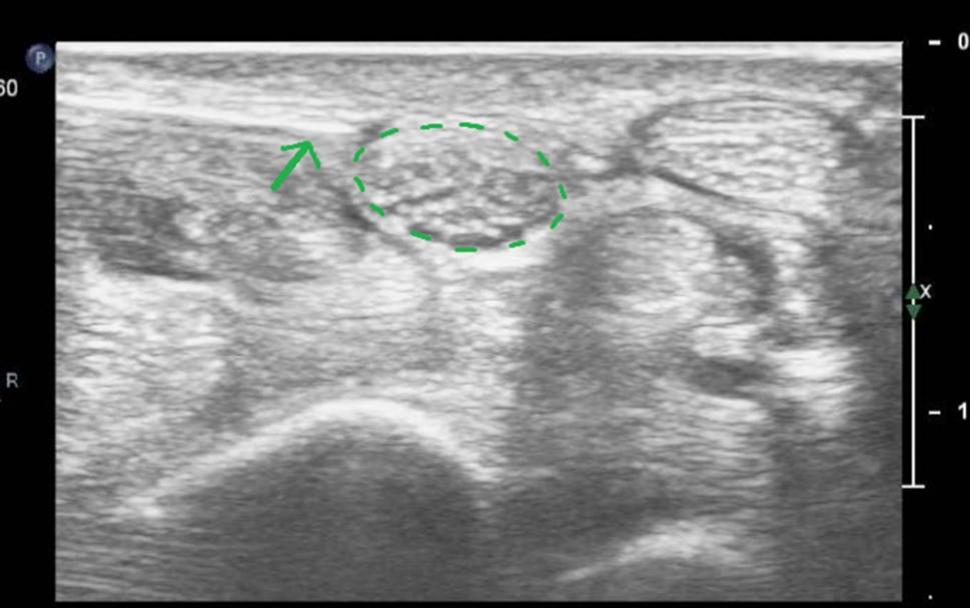
Ultrasound image of the carpal tunnel in the axial plane during Step 5 of the hydrodissection. *Legend:* circle in dotted line: median nerve; arrow: injection needle

**Fig. 5:**
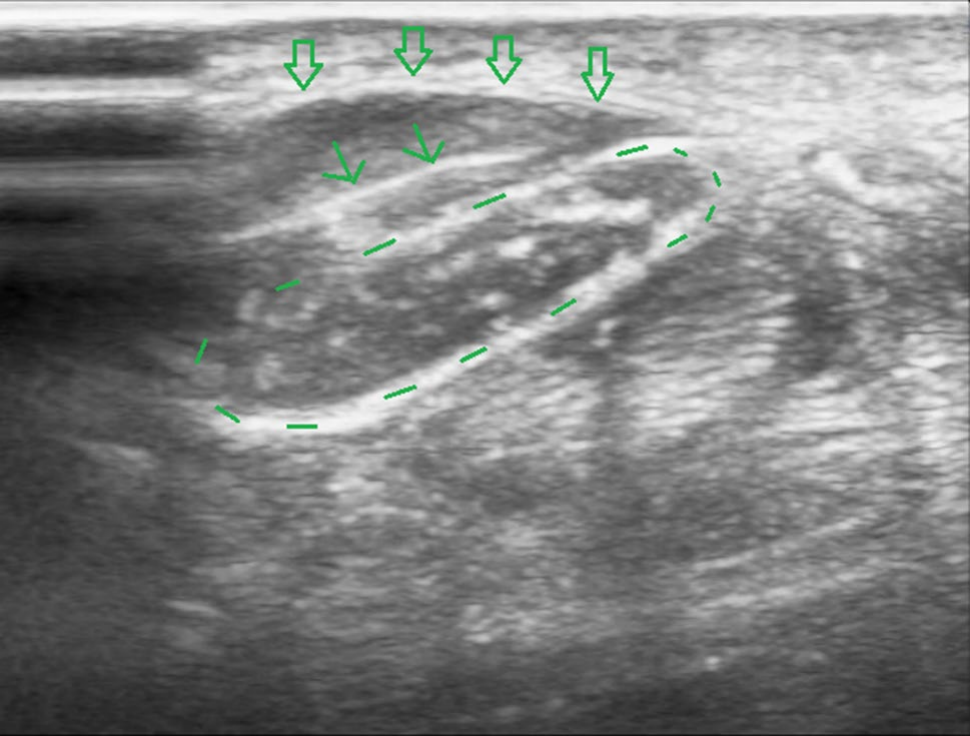
Ultrasound image of the carpal tunnel in the axial plane during Step 6 of the hydrodissection. *Legend:* circle in dotted line: median nerve; arrow: injection needle; void arrows: flexor retinaculum or transverse carpal ligament

**Fig. 6:**
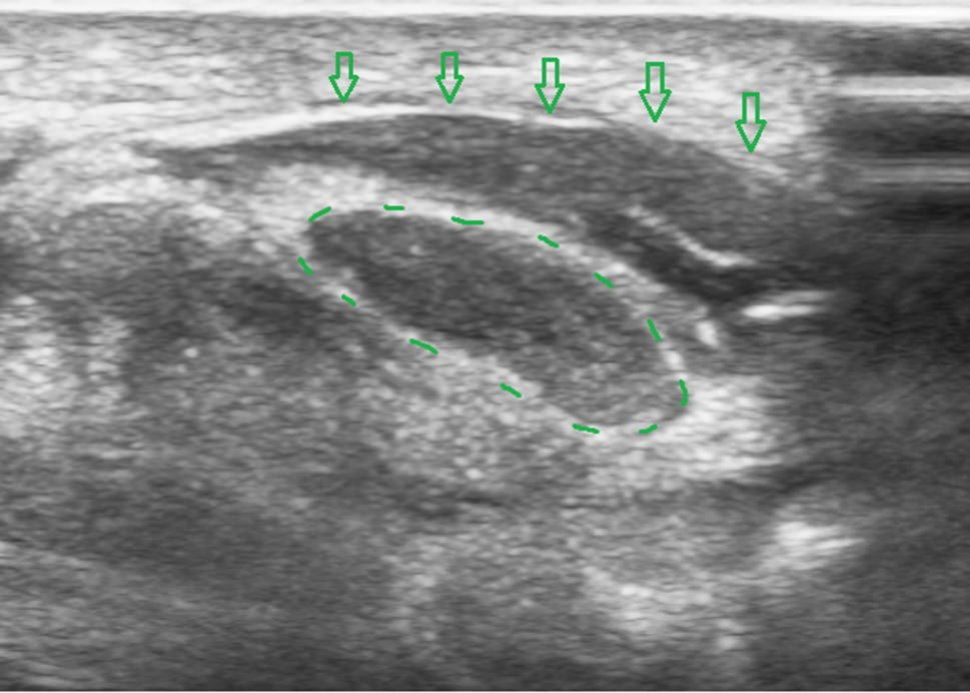
Ultrasound image of the carpal tunnel in the axial plane during Step 7 of the hydrodissection. The median nerve is fully dissected from the flexor retinaculum. The anechoic fluid (on this image a layer of approximately 4 mm) between the retinaculum and the median nerve is the injected solution. *Legend:* circle in dotted line: median nerve; void arrows: flexor retinaculum or transverse carpal ligament

1. Patients are seated or in a supine position, with the forearm supinated (slightly oversupinated) and the wrist placed in slight dorsiflexion with the use of a rolled-up towel (Fig. 1).
2. Sterilize from the mid-forearm to the entire palm with chlorhexidine 2%. Then apply a sterile covering on the ultrasound probe and a sterile gel on the skin.
3. Place a high-frequency ultrasound probe about 4 cm proximal to the wrist, oriented in the transverse (or, anatomically, axial) plane of the wrist. Move the transducer leisurely down until the carpal tunnel is reached, and then move the probe slightly ulnarly while keeping the median nerve in view. In this manner, the flexor retinaculum can be observed together with the median nerve (Fig. 2).
4. Insert a 27-gauge needle (0.40 × 40 mm) from an ulnar approach. Insert the needle just inferior to the deep surface of the median nerve. Be aware of the ulnar nerve and artery. The needle first approaches the inferior surface of the median nerve with the needle bevel positioned up. Inject a small volume of the solution here (Fig. 3); we injected 1.5 cc.
5. Pull the needle back up to just below the insertion point, turn the needle with the needle bevel positioned down, and then advance the needle to just below the retinaculum and ulnar of the median nerve. Slowly advance parallel with the skin until just ulnar to the median nerve (Fig. 4).
6. Start injecting the solution at this position and advance parallel with the skin to peel the median nerve off the overlying flexor retinaculum via hydrodissection (Fig. 5). Use a minimum of 3 mL of the injectable solution for this step.
7. Ensure the separation between the nerve and the retinaculum (see the ultrasound image in Fig. 6) and move the probe 1.3 cm proximal and distal to the insertion site. If the nerve is still attached to one of the deep layers of the retinaculum (even just a small part), repeat Step 6. When the median nerve is fully adhesiolysed from the retinaculum, the procedure is finished.

This minimally invasive hydrodissection technique can be performed with different solutions: anaesthetic, corticoids, saline, 5% dextrose, or even platelet-rich plasma (PRP). We used 1 mL corticoid solution (betamethasone dipropionate and sodium phosphate: 5 mg and 2 mg, respectively) diluted with 3.5 mL anaesthetic. The aim is to obtain a combination of the anti-inflammatory effect of the cortisone on the oedema and the inflammation of the nerve with the adhesiolysis of the retinaculum. In this article, we will not discuss the medication options further.

## Discussion

This study describes a new strategy using hydrodissection to manage CTS. The strategy is in essence an extension of the common ultrasound-guided injections but with an ulnar approach. This hydrodissection technique for CTS is minimally invasive and accessible to all physicians who are familiar with ultrasound-guided injections. The new approach involves one injection to hydrodissect the median nerve from the retinaculum. The needle is under direct, in-plane visualization during the entire procedure. Compared with other techniques, this drastically reduces the risk of complications, such as piercing the ulnar artery, the flexor tendons, or the median nerve itself [5, 6]. We consider our new technique to be safe, effective, accurate in placing the medication, and easy to perform. More than 30 procedures were performed since January 2020, and no complications have been reported to date. In the treatment of CTS, this new hydrodissection technique might become a feasible intermediate treatment modality between initial conservative measures and open release in CTS.

Classical carpal tunnel injections have been known for many years as a treatment modality for CTS. A widely used ultrasound-guided CTS injection technique is described by Smith et al. [7]. This technique includes an ulnar approach, transverse imaging of the carpal tunnel, long‐axis (in‐plane) imaging of the needle, and versatility in targeting structures within the carpal tunnel [7]. However, we expect better results with our new approach because, in addition to improved accuracy in medication placement, the new technique peels the median nerve off the retinaculum with a minimally invasive hydrodissection technique. Pressure and adhesions of the (thickened) flexor retinaculum on the median nerve may be a cause of failure of the classical injections [3]. Neuropathic pain can result from even minimal nerve compression. It supports the notion that the slightest compression can cause structural changes in nerves as well as neuropathic pain. A direct mechanical benefit from nerve release may result from the restoration of nervi or vasa nervorum function through the release of pressure [8]. The described hydrodissection technique disrupts the adhesions and the encircling of the superior portion of the median nerve.

**Fig. 7:**
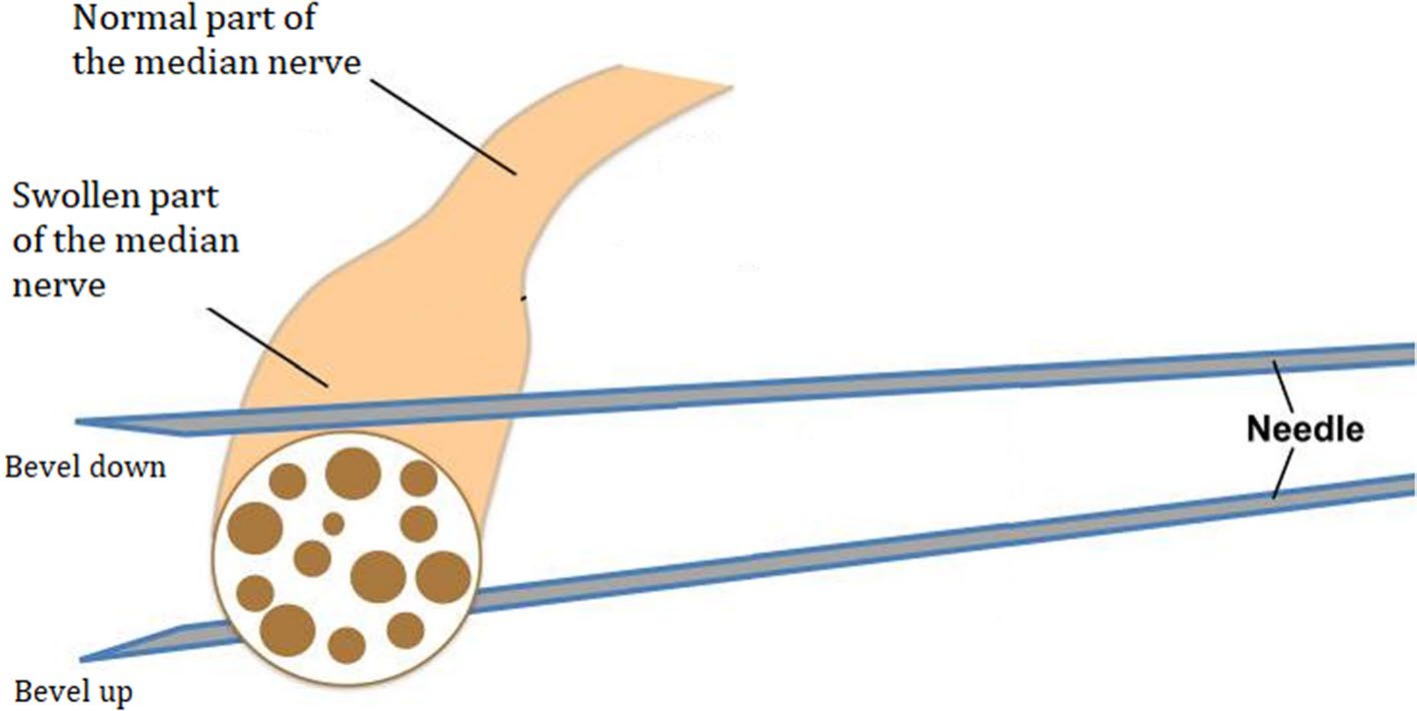
Illustration of the needle positions (bevel up vs. bevel down)

The first three steps of the new technique consist of positioning the patient and the work surface, correctly sterilizing the skin, and positioning the ultrasound probe (Fig. 1). The insertion of the needle starts in Step 4. First, the needle approaches the inferior surface of the median nerve with the needle bevel positioned up. The medication of the injectate is placed just beneath the nerve (Fig. 3). It is important to approach the inferior surface of the nerve with the bevel up to avoid piercing the median nerve because the resistance of the soft tissue will generally force the needle to go deep [3]. In Step 5, when the needle is withdrawn until the subcutis, the needle is turned with the bevel positioned down. The injectate will separate the median nerve from the retinaculum while advancing the needle (Step 6) (Figs. 4, 5). Throughout this peel-off procedure, it is important to keep the needle with the bevel down so that the resistance of the soft tissue will force the needle to move more superficial to avoid piercing the superior part of the median nerve (Fig. 7) [3]. After Step 6, the median nerve appears oval and surrounded by the anechoic fluid on the ultrasound image (Fig. 6). When this image is obtained, all the adhesions of the median nerve and retinaculum are released, and the hydrodissection is complete.

The current study has some limitations. Although we performed more than 30 procedures, no prospective or retrospective study with long-term results and side effects was conducted. This article aims to introduce the new technique of minimally invasive hydrodissection for CTS and to explain the approach in detail. In this manner, we give colleagues the opportunity to use this procedure in practice and, if possible, perform further studies on this new technique.

## Conclusion

Although the hydrodissection technique is an upcoming technique that is mentioned in several articles, no protocol has yet been described in the literature for a safe and easy-to-use hydrodissection technique for CTS. We describe a new approach that involves one injection to be performed with a minimally invasive procedure. The needle is under direct, in-plane visualization during the entire procedure. The new technique does not require injecting a large volume of solution (> 10 mL) as previously described. We describe our technique in this article, but no retrospective or prospective study was conducted. Although the technique seems promising in terms of safety and effectiveness, retrospective and randomized controlled trial studies are needed for this approach to be regarded as a feasible intermediate treatment modality between conservative treatments and open release.
